# Exploring the dark foldable proteome by considering hydrophobic amino acids topology

**DOI:** 10.1038/srep41425

**Published:** 2017-01-30

**Authors:** Tristan Bitard-Feildel, Isabelle Callebaut

**Affiliations:** 1 CNRS UMR7590, Sorbonne Universités, Université Pierre et Marie Curie – Paris 6 – MNHN – IRD – IUC, Paris, France

## Abstract

The protein universe corresponds to the set of all proteins found in all organisms. A way to explore it is by taking into account the domain content of the proteins. However, some part of sequences and many entire sequences remain un-annotated despite a converging number of domain families. The un-annotated part of the protein universe is referred to as the dark proteome and remains poorly characterized. In this study, we quantify the amount of foldable domains within the dark proteome by using the hydrophobic cluster analysis methodology. These un-annotated foldable domains were grouped using a combination of remote homology searches and domain annotations, leading to define different levels of darkness. The dark foldable domains were analyzed to understand what make them different from domains stored in databases and thus difficult to annotate. The un-annotated domains of the dark proteome universe display specific features relative to database domains: shorter length, non-canonical content and particular topology in hydrophobic residues, higher propensity for disorder, and a higher energy. These features make them hard to relate to known families. Based on these observations, we emphasize that domain annotation methodologies can still be improved to fully apprehend and decipher the molecular evolution of the protein universe.

The protein universe is a concept corresponding to the set of all possible protein sequences that can be found in all living organisms^1^. Understanding how the current protein universe appeared and evolved are central questions in evolutionary molecular biology^2^,^3^,^4^. Moreover, deciphering the rules behind the apparition of new proteins and their conservation in an organism may lead to develop improved methods for protein design. The protein universe cannot be explored exhaustively in an expert-based approach, due to the huge amount of data available today. However, the development of powerful bioinformatics methods gave the opportunity to automatically classify and annotate protein sequences. The task is challenging but the community effort to give access to all protein sequences led to the fast emergence of several resources for protein annotation that are still intensely maintained up-to-date^5^,^6^,^7^,^8^.

It is well established that domains, *i.e*. those basic units from which proteins are made, are the essential blocks of protein evolution^4^,^9^,^10^. Two distinct definitions of a protein domain co-exist regarding the type of molecular data used for their identification. If structural data are considered, a protein domain can be defined as a basic unit that has the ability to fold independently and is characterized by an overall compactness, driven by the presence of a hydrophobic core, or by that of metal ions or disulfide bridges^11^,^12^. If the protein sequence information is used, a protein domain then corresponds to the amino acid sequence part that is evolutionary conserved and for which similar sequences can be found in other proteins of the same or in different organisms^8^. In the present study, references to protein domain will be based on this last, sequence-based definition, as it allows the largest coverage of the protein sequence space. In this sequence-based definition, the foldability is an optional characteristic; however, there is a good general agreement between the two types of domain definitions, as assessed by the comparison of sequence-based and structure-based domain databases^13^,^14^.

Several protein domain databases^5^,^6^,^7^,^8^,^15^ have been developed allowing automatic protein annotation. They give a broad view of the evolutionary mechanisms at play in the emergence, disappearance and re-use of the evolutionary units that are protein domains. The problems of such a methodology are the finite number of domains available for annotating a protein sequence and the limitation in the choice of the initial protein sequences that are used to define a domain family. Statistical models such as hidden markov models (HMMs) or position-specific scoring matrices (PSSMs) are usually built on a protein domain alignment seed, and this seed is afterward used to find occurrences of the domain in other protein sequences. Software and databases using domain HMMs/PSSMs can achieve great sensitivity and are able to annotate protein sequences sharing less than 20% of similarity^16^. However, the initial set of seeds constraints the direction that the annotation can take and some parts of the sequences of the protein universe remain without annotation, particularly in non-model organisms, without being however true orphan sequences. Even well characterized organisms such as *Homo sapiens* or *Escherichia coli* have a part of their proteome in the dark^17^, *i.e*. protein sequences without annotations. For instance, Pfam-A coverage per residue is limited to only 45% of the human proteome even if a large portion of human proteins, 90%, has at least one Pfam annotation^17^.

Part of the protein universe that remains without annotation is referred to as the dark matter of protein universe, or dark proteome, in reference to the seminal definition given by Michael Levitt in 2009^1^, and received much attention^18^,^19^,^20^,^21^,^22^ as this may include biological systems that are not yet known. In their recent work, Perdigão and colleagues^23^ made a distinction between proteins that are fully without annotation, called dark proteins, and segments of proteins without annotation, named dark regions^21^. However, they limited the annotation of the “known” universe to regions that are covered by known 3D structures or by models derived from structural information (named gray regions). They then focused on the properties of the dark proteins, highlighting that only a part of them corresponds to intrinsic disorder and transmembrane regions.

The originality of the present study is: (i) to enlarge the definition of the known universe by considering sequence-based domain databases (for the description of the gray regions, as introduced by Perdigão and colleagues^23^) and (ii) to analyze part of these un-annotated sequences that correspond to foldable domains. These last domains are currently difficult to isolate in absence of homologous sequences, *i.e*. without the knowledge of domain models, a fact that explains their absence in domain databases. The detection of un-annotated, but foldable domains of the dark proteomes relies on the use of an automatic tool, called SEG-HCA, derived from the Hydrophobic Cluster Analysis (HCA) methodology. It allowed to identify, in a comprehensive way, foldable domains from the only information of a single amino acid sequence, without the prior knowledge of homologous sequences^14^. The principle of this approach, whose reliability has been supported by confrontation to both structure and domain databases^14^, is to delineate regions characterized by a high density in hydrophobic clusters, which mainly correspond to regular secondary structures that form globular domains^24^,^25^. The properties common to these un-annotated foldable domains were then analyzed, in order to understand what let them in the dark. To this purpose, the un-annotated foldable domains were compared to known domains annotated using two distinct methodologies, considering domains with structural homologs detected by mapping of the Protein Data Bank (PDB)^26^ as well as protein domain families stored in databases. As in the work of Perdigão *et al*.^23^, domain annotations were performed on the Uniprot/Swissprot dataset^27^. The quality of the sequences from this dataset has indeed the advantage to strongly limit the potential bias from artefacts coming from genome annotation assembly or prediction errors, in addition to provide a balanced set of proteins from different organisms, thus avoiding over-representation of some taxa. This high quality dataset thus supports the relevance of the differences observed between un-annotated foldable domains and known domains stored in databases. Some differences are striking, in particular at the level of the hydrophobic amino acids topology. Finally, several remote homology detection tools were used to estimate the proportion of the un-annotated domains of the dark proteome that can be related to known folds and highlight those that may correspond to true novelties. Interestingly, the less information there is for an un-annotated domain the more important is the difference at the level of the hydrophobic amino acid topology relative to known domain families.

## Results

### The dark proteome, and the importance of the methodology used for annotation

Starting from an initial set of 549 832 proteins extracted from Uniprot/Swissprot, different methods of annotations were used and led to distinguish between annotated protein segments and protein segments or full protein sequences that weren’t annotated. These last sequences correspond to the dark protein universe, *i.e*. the unknown parts of the protein universe, by reference to the works of Levitt^1^ and Perdigão *et al*.^23^. Why these sequences are un-annotated and to which kind of molecular units they correspond, if they do, are unanswered questions that could bring novel insights into our understanding of the evolutionary dynamics of the protein universe.

In this study, four categories were used to distinguish different types of annotations: PDB regions (sequences for which experimental structural data are available), gray regions (automatic domain annotation), dark regions and dark proteins. Dark regions are defined as residues that are not assigned to a PDB entry (Uniprot/PDB mapped regions) or to a protein domain model. A protein is considered as a dark protein if a single dark region covers the entire protein sequence. Gray regions include information coming not only from structural source (Protein Model Portal), as in the work of Perdigão *et al*.^23^, but also from sequence sources (Pfam and CDD HMMs). This should thus significantly extend the coverage of the protein universe by known domains. However, it is important to note in this context that the structural information (with or without PMP annotations) is well covered by domain profiles (93.12 and 78.86%, respectively), indicating that domain annotations well capture the key features of folded domains. In contrast, coverage of the gray regions by structural information (PMP and Uniprot/PDB mapped regions) is 64.02%, and only 3.11% when PDB is considered alone for annotation, clearly highlighting the enlargement of relevant information that can be made using domain profiles.

Figure S1 illustrates the percentages of amino acids belonging to each of the four categories (dark proteins, dark regions, gray regions and PDB regions). The percentage of amino acids resulting from a direct mapping between the PDB and Uniprot is relatively small in all four kingdoms (Eukaryota (E): 3.81%, Bacteria (B): 1.96%, Archaea (A): 4.63%, Viruses (V): 3.43%), highlighting the relatively small part of the protein universe for which three-dimensional structures have been solved. The amount of dark proteins, *i.e*. percentage of amino acids of proteins without any annotation, is of the same order of magnitude (E: 1.61%, B: 0.33%, A: 1.26%, V: 8.74%). As in the study of Perdigão and colleagues^23^, a larger proportion of dark proteins is detected in the viral protein datasets than in the other three kingdoms. This is in agreement with the fact that viral proteins are known to be difficult to annotate, by contrast with the annotations found for bacterial and archaeal proteomes. The largest part of the amino acid sequences in the Uniprot database is made of gray regions, *i.e*. parts of protein sequences annotated by at least one domain model (E: 57.23%, B: 92.84%, A: 89.46%, V: 54.49%). The remaining sequences constitute the dark regions of the protein universe, *i.e*. un-annotated parts of proteins that have annotation elsewhere on their sequences (E: 37.35%, B: 4.87%, A: 4.64%, V: 33.33%).

These results slightly differ from those presented by Perdigão and colleagues^23^ (dark protein coverage: 15%, 5%, 6% and 28% for Eukaryotic, Bacterial, Archaeal and Viral sequences, respectively, and dark region coverage: 29%, 8%, 8%, 26% in the same order). The larger coverage of the gray regions in our analysis can be attributed to the use of HMMs for delineating protein domains, which greatly improves proteome annotation. Of note is that the decrease of dark annotation in our study is more pronounced for dark proteins, rather than for dark regions.

### The dark foldable proteome: delineation and characterization

A search for potential “foldable” domains in the sequences of the dark universe was performed using SEG-HCA^14^, a tool recently developed and based on the Hydrophobic Cluster Analysis (HCA) approach^14^,^25^,^28^,^29^,^30^. HCA hydrophobic clusters, made of strong hydrophobic amino acids (V, I, L, F, M, Y, W)^31^,^32^, are different from hydrophobic segments as they can incorporate other, non-hydrophobic, residues. This property originates from the use of a two-dimensional alpha-helical net, resulting in connecting hydrophobic amino acids separated by up to three non-hydrophobic amino acids (or a proline). Hydrophobic clusters defined in this way (with this hydrophobic alphabet and the connectivity distance associated with the α-helix) have been shown to match at best regular secondary structures (α-helices and β-strands) and to constitute hallmarks of folded domains^24^,^25^. The principles of the method are recalled in supporting Fig. S13 of the Supporting Information (Method section). SEG-HCA segments, which correspond to regions where a high density in hydrophobic clusters is detected, have been shown to correspond to domains which have the ability to fold, either in an autonomous way or following contact with partners^14^,^33^; these segments are later referred to as HCA domains. The advantage of SEG-HCA in the characterization of the dark proteome is to allow the prediction of these foldable domains from the only information of a single amino acid sequence, without the prior knowledge of homologous sequences.

In the remaining of the manuscript, references to sequences of the four groups (PDB regions, gray regions, dark regions and dark proteins) correspond to HCA domains of these groups, except if specified otherwise, as the goal of this study is to understand why these foldable domains are not annotated, in contrast to globular domains stored in domain databases. Short HCA domains, less 30 amino acids long (21.8% of the total number of HCA domains), were also discarded to only keep HCA domains with a high trend toward foldability.

The amino acid coverage by HCA domains is presented in Supporting Information Fig. S2 and Table S1. The HCA domains well cover regions classified as PDB and gray as they correspond to annotated foldable domains. The ratio of covered versus uncovered HCA domains is less important for sequences from the dark regions and the dark proteins. However, a large portion of these dark sequences (75.78% and 83.24% of amino acids from dark regions and dark proteins respectively) are still covered by HCA domains and are likely to correspond to foldable sequences.

A comparison of the lengths of HCA domains between the dark regions/dark proteins and the gray regions/PDB regions is shown in Fig. S3. HCA domains in the gray regions (mean length of 195 amino acids) and in the PDB regions (mean length of 174 amino acids) have similar length distributions, while dark proteins have a higher amount of shorter HCA domains (mean length of 130 amino acids). Dark regions exhibit the shortest HCA domains (mean length of 114 amino acids). Even if their mean length is smaller than in the other categories, the HCA domains from the dark proteomes are long enough to be able to fold. Furthermore, the modes of these distributions are similar for domains from PDB regions, gray regions and dark protein sequences (around 60 amino acids) and a little smaller for the dark region sequences (around 50 amino acids).

**Figure 1:**
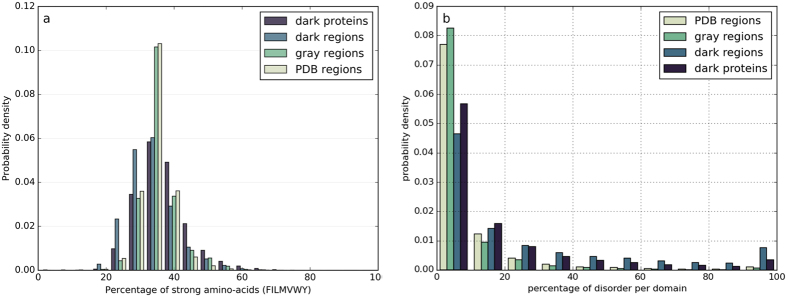
Hydrophobicity and disorder (HCA domains of the protein universe). (**a**) Distributions of the frequencies in strong hydrophobic amino acids (V, I, L, F, M, Y, W) of foldable (HCA) domains in the different groups of protein sequences. (**b**) Distribution of disorder coverage within foldable (HCA) domain sequences.

The compositions in amino acids do not differ significantly between the different categories (Fig. S4), with a maximum fold change (computed as the ratio of two quantities minus 1) in the mean amino acid percentage observed for serine between the sequences of the PDB regions and of the dark regions (fold change of 0.36). The amino acid content per sequence also appears to have a large spectrum of values inside each group (Fig. S5). However, the distributions of amino acids percentages considering all strong hydrophobic amino acids (V, I, L, F, M, Y, W; Fig. 1a) show clear differences between the four groups of sequences (two sample Kolmogorov-Smirnov tests, p-values ≪ 0.001), the dark proteins and dark regions being characterized, on average, by a lower content in strong hydrophobic amino acids but also a larger variance. Interestingly, sequences of dark regions and dark proteins also have more short and long hydrophobic clusters, respectively, than the other groups (Fig. S6), pointing out a different organization at the hydrophobicity/secondary structure level. The amount of predicted intrinsic disorder was calculated for the HCA domains of the four categories using the consensus data from the MobiDB database^34^. Differences are again observed for HCA domains of the dark proteins and dark regions, these having on average a higher content in predicted disorder (Fig. 1b) (two sample Kolmogorov-Smirnov tests, p-values ≪ 0.001). This trend is also observed for the prediction of regions undergoing folding upon binding by the ANCHOR program^35^,^36^ (Fig. S7a) (p-values ≪ 0.001).

The percentage of amino acids in transmembrane regions was also calculated for the HCA domains belonging to the four different groups: PDB regions include 0.58% of amino acids predicted in transmembrane segments, gray regions 3.34%, dark regions 2.01% and dark proteins 3.59%. As expected, domains in PDB regions display a lower coverage. Interestingly, the percentage of residues belonging to transmembrane segments is very similar between HCA domains of gray regions and dark proteins. Their percentages of residues annotated as part of transmembrane regions are the only ones above 3%. This indicates that the percentage of transmembrane regions from domain databases is similar to the percentages found in domains delineated by SEG-HCA.

**Figure 2:**
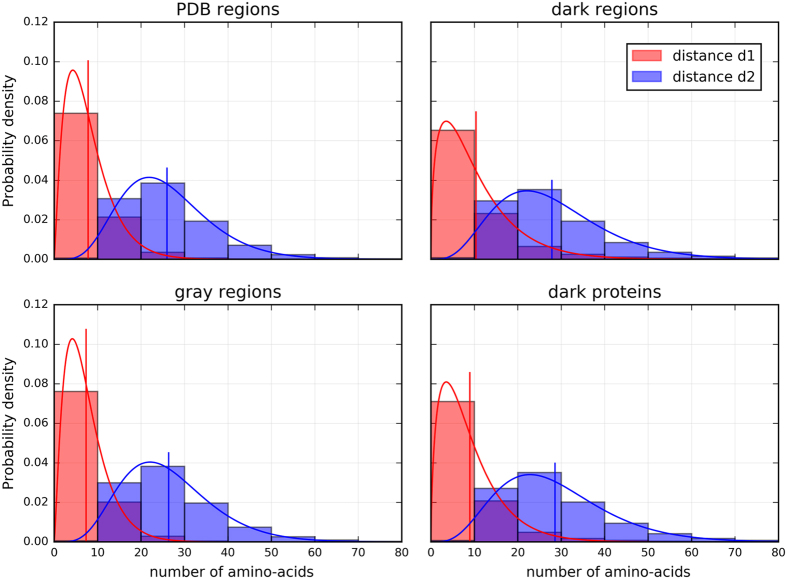
Distances between hydrophobic clusters (HCA domains of the protein universe). The distance d1 corresponds to the number of amino acids between two hydrophobic clusters, the distance d2 corresponds to the distance d1 plus the number of amino acids within the two hydrophobic clusters (see Fig. S12). Distributions were fitted using a gamma distribution (solid lines), the vertical lines corresponding to the expected values of the fitted distributions.

Further analyses were made by calculating the distances between hydrophobic clusters included in HCA domains of the four categories. The first distance, called d1, is the distance between the last residue of a hydrophobic cluster and the first residue of the following one, and the second distance, called d2, is the distance between the first residue of a first hydrophobic cluster and the last residue of the second one (see Fig. S13 in the Supporting Information). Figure 2 shows very similar distributions of distances for the sequences from the PDB and the gray regions. In contrast, a small shift toward longer distances is observed for the distribution of the sequences of the dark proteins and the dark regions, for both the d1 and d2 distances, being even more pronounced for d1. This indicates that hydrophobic clusters are separated by larger sequence segments. The shift is not a consequence of the difference in domain length, as supported in Fig. S8 showing the d1 and d2 values for random sequences (amino acid frequencies computed from the PDB) and where it can be observed that larger sequences have longer distances than shorter ones. In our case, sequences of HCA domains from dark regions and dark proteins are shorter than sequences from gray regions and PDB regions but are characterized by longer d1 and d2 distances. Therefore, this effect is most likely interpreted as a difference in topological organization of hydrophobic clusters (two sample Kolmogorov-Smirnov tests, p-values ≪ 0.001).

**Figure 3:**
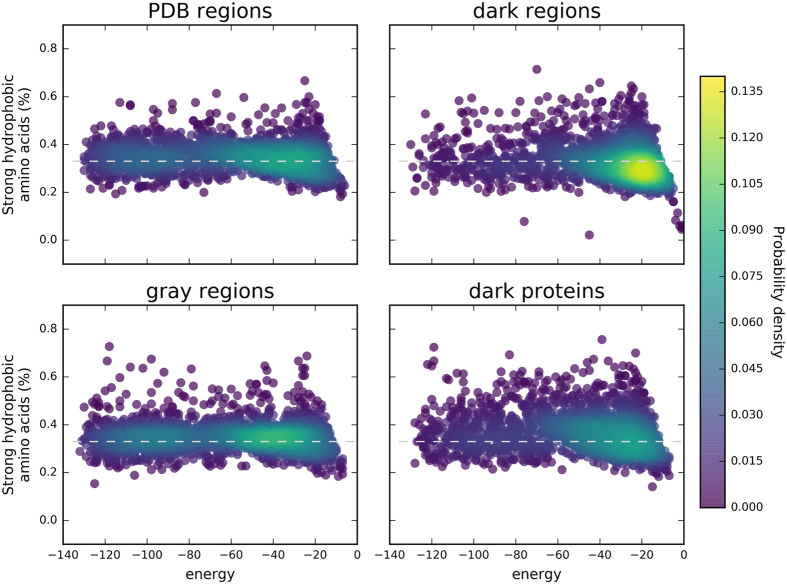
Frequency in strong hydrophobic amino acids (V, I, L, F, M, Y, W) and protein sequence energy (HCA domains of the protein universe). Frequency and energy were computed for the different groups of sequences. The energy corresponds to the folding energy computed using HP-models (HPstruct). The colormap corresponds to the probability density computed from a Gaussian kernel estimation over the data points.

Owing to their definition, HCA domains have a high potential toward foldability. Figure 3 shows the relation between the number of strong hydrophobic amino acids in a protein sequence and the level of energy computed in a HP-lattice model for 5000 protein domains randomly chosen within the different groups considered here. The sequences of the PDB regions appear to have less variability in terms of frequency in strong hydrophobic residues, which is around 0.33, corresponding to the standard frequency of hydrophobic amino acids observed in globular proteins. A high density of sequences can be observed at the center of the distribution, with an energy level around −60. This energy basin shifts toward higher energy levels for the sequences of the three other groups. Moreover, contrary to the sequences of the gray regions for which the ratio of 0.33 in strong hydrophobic amino acids is maintained, sequences from the dark proteome groups have a much more dispersed distribution. A difference in the energy basin is also observed between the sequences of the dark regions and those of the dark proteins. A high density of sequences is observed below the 0.33 hydrophobic ratio limit for dark regions, while for comparable levels of energy, the sequences of the dark proteins form a high density region at the 0.33 limit. The comparisons between the energy distributions of the four categories led to significant differences (two sample Kolmogorov-Smirnov tests, p-values ≪ 0.001).

In conclusion, the higher content in predicted disorder, the smaller content in strong hydrophobic amino acids and the larger d1 and d2 distances between hydrophobic clusters indicate that a large proportion of HCA domains of dark sequences display a less organized behavior (in terms of regular secondary structure density) than their PDB and gray counterparts. The differences between energy distributions regarding the hydrophobic ratio of each group are in agreement with this result. This behavior doesn’t correlate with full disorder of the dark proteome as Perdigão and colleagues^23^ reported previously, as the sequence of dark proteome is characterized by various degrees of disorder. Moreover, our results are reflecting the continuum of the protein sequence space universe ranging from fully compact proteins to fully disordered proteins. The sequence organization of the different annotation groups (PDB regions, gray regions, dark regions, dark proteins) correlates with different degrees of the protein sequence universe disorder, in agreement with previous studies on intrinsically disordered proteins^37^,^38^.

### Going further into the dark regions

To remove the potential noise in our characterization of HCA domains that should result from undetected similarities due to the limited sensitivity of domain database profiles, PSI-BLAST searches were first performed using HCA domains of the dark proteome as queries, against the sequences of the gray and PDB regions. Queries were then removed in case of an alignment with another sequence, with an E-value lower than 1e10^−3^ and a coverage of the HSPs superior or equal to 50% of the query length. From the initial 223912 HCA domains, 67684 were filtered out in this way. These 67684 HCA domains mainly correspond to domains from dark region sequences (97.36%), the remaining ones (2.64%) corresponding to domains from dark proteins.

The degree of redundancy in HCA domains was then analyzed using psi-cd-hit, in order to cluster similar sequences of the dark proteome. The results, obtained by using a sequence identity threshold of 30%, are displayed in Table 1. Interestingly, 67217 out of the 156228 sequences (43.02%) in the dark proteome dataset don’t have any obvious similarity with any other sequence and 51972 others (33.27%) are in clusters containing between 2 to 4 sequences. Moreover, 73.71% of the clusters containing more than one protein are made of proteins with the same Uniprot family identifier, whereas only 18.17% of the clustered proteins are made of two different names (99% of all clustered sequences are made of 1 up to 6 Uniprot family identifiers). This indicates that a large amount of HCA domains are consistently present in the different members of a same family.

Altogether, these results indicate that most of the sequences don’t share any obvious similarity with other sequences, thus behaving as true orphan sequences. When HCA domains cluster together, they also usually belong to the same Uniprot family. Inside these clusters, 1538 HCA domains from dark proteins (10.89% of the number of dark protein segments used as psi-cd-hit queries) were part of a cluster also possessing an HCA domain from dark regions. Moreover, a large part of these sequences (50.70% of the total number of clusters) are of relative small length (<60 amino acids).

As the sequences clustered by psi-cd-hit have to some extent some similarity relationship, the remaining sequences, *i.e*. HCA domains that are not in clusters, were selected for further analyses. This set of 67 217 orphan HCA domains comes from dark regions (59 075 sequences) and dark proteins (8142 sequences).

**Figure 4:**
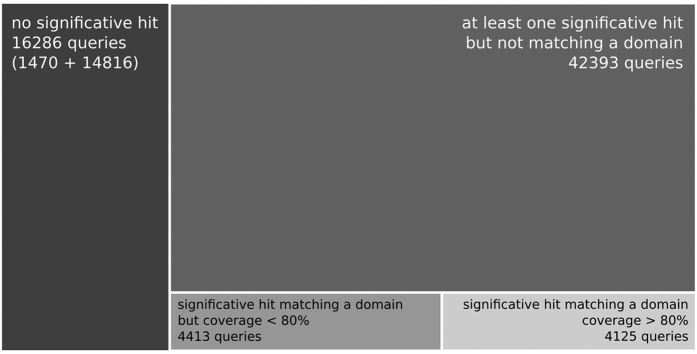
Repartition of HCA domains from the dark proteome according to the TREMOLO-HCA results. The sizes of the rectangles are proportional to the number of protein domains.

Further analyses on these orphan HCA domains were performed using TREMOLO-HCA, which allows the detection of remote relationships in similarity search results (here performed using HHblits^39^), based on the consideration of fold signatures as well as of the domain architecture of the compared sequences (assigned by using CDD)^30^. Figure 4 displays the repartition of the orphan HCA domains according to the TREMOLO-HCA results. 1470 HCA domains, out of these 67 217 sequences, don’t match any target sequence in the TREMOLO-HCA results and 14 816 HCA domains have at least one, but not significant match. According to these results, 16 286 HCA domains thus belong to the deep dark proteome universe. 4125 queries significantly match a domain from a target sequence in the TREMOLO-HCA results (e-value for the target’s domain alignment <0.001, HHblits e-value of hit <0.001 and domain coverage ≥ 80%), 4413 others HCA domain queries don’t reach the 80% coverage threshold but have at least one significant match with an annotated domain (HHblits e-value < 0.001, target’s domain alignment e-value < 0.001 and domain coverage >0% but <=80%). Finally, 42 393 HCA domains don’t match any annotated domain (for the target’s domain alignment e-value > 0.001) but have a significant match with a target sequence in the TREMOLO-HCA result (HHblits e-value of hit <0.001). These last HCA domains were clustered together using MCL on a graph created with nodes and edges corresponding to the HCA domain queries and the number of common matches between HCA domains. Such clustered HCA-domains based on common targets allowed us to identify case study of potential hidden domain families.

**Figure 5:**
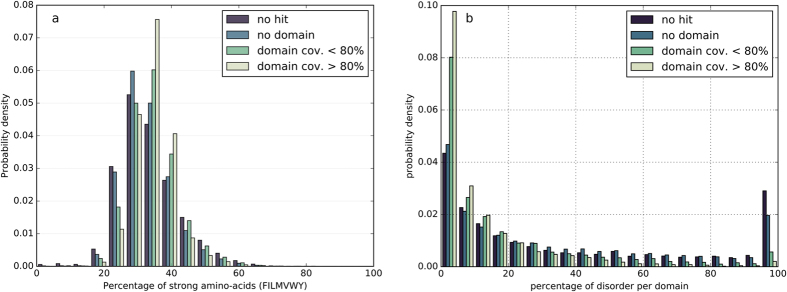
Hydrophobicity and disorder (dark sequences after TREMOLO-HCA filtering). Distributions of the frequencies in strong hydrophobic amino acids (**a**) and intrinsic disorder (**b**) of the foldable domains, classified according to their similarity to other domains, as defined using the TREMOLO-HCA results.

As reported above, the four initial groups of protein sequences (PDB regions, gray regions, dark regions, and dark proteins) displayed significant differences in their hydrophobic and intrinsic disorder contents. These two properties were re-estimated for the four groups of sequences from the dark proteome delineated on the basis of the TREMOLO-HCA results. Figure 5a shows the distribution of the percentages in strong hydrophobic residues per protein sequence. As for the dark regions and dark proteins sequences, TREMOLO-HCA-defined groups with the weakest annotations, corresponding to HCA domains without any hit or with hits not matching known domains, display a shift of the distribution of the strong hydrophobic residues content to lower values (two sample Kolmogorov-Smirnov tests, p-values ≪ 0.001). HCA domains with more annotations, either fully or partially associated with an annotated domain, have a distribution in strong hydrophobic residues similar to the initially annotated sequences, from PDB regions or gray regions. Figure 5b shows the intrinsic disorder for the same TREMOLO-HCA clustered groups and Fig. S7b shows the distribution of sites with disorder-to-order upon binding predicted by ANCHOR. The HCA domains groups, without HHblits hits or with HHblits hits not associated with already annotated domains, show a higher tendency to intrinsic disorder than the HCA domains groups to which an annotated domain can be linked (two sample Kolmogorov-Smirnov tests, p-values ≪ 0.001).

The percentages of sequence coverage by putative transmembrane regions were also computed 1.74% and 1.85% of the amino acids of HCA domains matching an annotated domain (with a query to target coverage >80% and <=80% respectively) correspond to putative transmembrane regions. These percentages are 1.62% and 2.66% for HCA domains without match to an annotated domain and without significant hit, respectively. They are of the same order of magnitude than the percentages computed for the domains found in PDB regions, gray regions, dark regions and dark proteins, indicating the absence of enrichment in transmembrane regions in the HCA domains selected through the remote similarity methodologies used.

**Figure 6:**
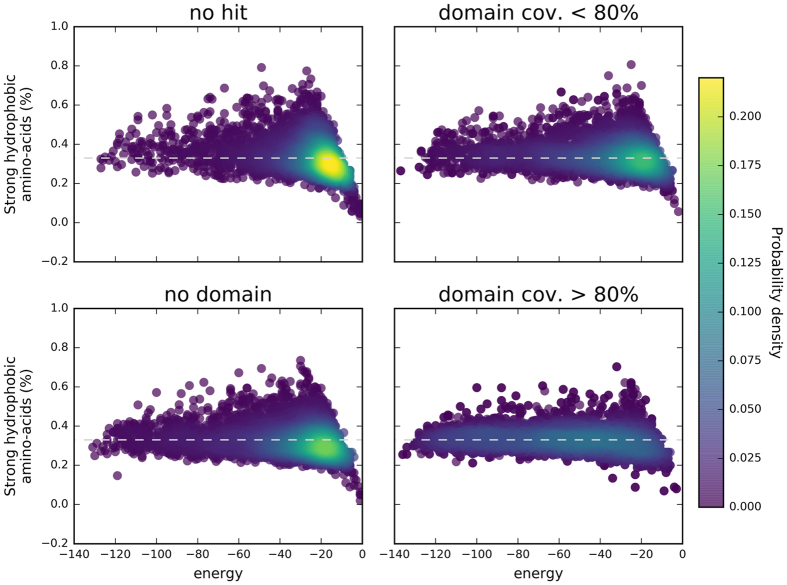
Frequency in strong amino acids (V, I, L, F, M, Y, W) and protein sequence energy (dark sequences after TREMOLO-HCA filtering). Frequency and folding energy (HP-struct) were computed for the four final groups of HCA domains from the dark proteomes after Tremolo-HCA filtering. The colormap corresponds to the probability density computed from a Gaussian kernel estimation over the data points. The four groups of HCA domains have distributions similar to those obtained with the initial groups of sequences (dark proteins and regions, gray regions, PDB regions).

Interestingly, as observed in Fig. 6, the folding energies, estimated using HP-lattice models, of the four different dark groups defined on the basis of the TREMOLO-HCA results display a pattern similar to the four groups of initially classified sequences (PDB regions, gray regions, dark regions, and dark proteins). Indeed, dark HCA domains matching known domains have similar spectra of energy values as the sequences from PDB and gray regions. Dark HCA domains partially matching a known domain already display values centred toward higher energy levels. The shift of energy levels toward higher values become stronger for dark HCA domains for which at least a significant hit was found, although not corresponding to an annotated domain. And finally, the HCA domains without any significant hit have the strongest signal for higher energy levels, similarly to the whole set of sequences of dark regions. The comparisons between the energy distributions of the four categories led to significant differences (two sample Kolmogorov-Smirnov tests, p-values ≪ 0.001).

Finally, for illustration purposes, we extracted examples of foldable domains of the dark proteome found in each of the categories depicted in Fig. 4 and show the corresponding HCA plots in Figs S9, 10, 11 and S12.

Figure S9 gives an example of a hidden BRCT domain. In this case, the dark HCA domain query sequence (fission yeast MDB1, Uniprot O14709, between amino acids 486 to 598) directly matched BRCT domains of targets from the uniprot_20 database, therefore, corresponding to the first group described above. The BRCT domain was easily recognized with its typical signature^40^ and can be aligned with the second BRCT domain of the tandem repeat of MDC1, its human ortholog, which is a key factor in DNA damage response^41^.

Figure S10 shows one of the 4413 dark HCA domain queries partially matching an annotated domain. The annotated domain corresponds to spectrin repeats (cd02488), which are small independent folding units forming triple coiled-coils^42^. Protein repeats largely belong to the dark matter of proteomes, as assessed by a study of the Pfam coverage of the human proteome, in which tandem repeats were found to fall into the less characterized clusters of protein sequences^17^. In fact, tandem repeats evolve quickly while maintaining their fold, rendering their detection difficult by traditional methods. This represents a challenging issue in structural bioinformatics^43^, that the methodology proposed here may help to solve.

Figure S11 highlights the presence of a newly detected domain found in two dark HCA domain queries (from proteins Sp100 and Sp140) and for which a similarity relationship is proposed based on their common targets in the TREMOLO-HCA results after the MCL clustering. The signature of the domain, which focuses on two conserved hydrophobic clusters (designated A and B) can be found in several target sequences and doesn’t correspond to any known fold in any of these target proteins. However, a precise amino acid sequence alignment is here hard to define, as this small domain appeared to have evolved quickly and to lack a strong hydrophobic core. Such a proposed relationship thus remains to be supported at the experimental level.

The last cases in Fig. S12 presents domains for which no information was found, *i.e*. dark HCA domains that didn’t show any significant sequence similarity with any other protein target and therefore correspond to true dark sequences.

## Discussion

The dark proteome constitutes a terra incognita of our understanding of the protein universe. It is a potential reservoir of new folds and new functions, and a challenging target for researchers whose goal is to elucidate the evolutionary relationships between actual protein sequences. Fully understanding these relationships will shed light on the evolutionary path leading to the current protein universe^1^,^3^,^44^,^45^. Furthermore, the annotation of protein sequences with the most complete list of domains as possible is now a necessary step in comparative analysis of species proteomes, as the gain, loss and expansion of protein domain families inform on the evolutionary history of the studied species^46^,^47^.

In this study, the Uniprot/Swissprot sequence database was thoroughly analysed using various tools to annotate domains, in order to provide the deepest annotation coverage of protein sequences. The influence of resources used for automated domain annotation of protein sequences was strongly emphasised. A variety of resources is needed to overcome the presence of potential bias in the set of initial domains used as models, and resources such as CDD^5^ or Interpro^6^ should be considered for comprehensive domain-based analyses. The relevance of some of the current classical domain databases was acknowledged, as they contributed to 73.51% (computed from our dataset) of the overall sequence annotations (excluding overlapping annotations with PDB and PMP databases). The segments of proteins, or entire proteins, without any annotation are not uniformly distributed between kingdoms; eukaryotic and viral sequences especially possess a large reservoir of un-annotated domains, making them interesting targets for further investigations.

Based on the protein sequence annotations, parts of the sequences were separated into four categories according to the domain annotation source: PDB regions (sequences annotated from direct structural information), gray regions (sequences annotated from domain family information), dark regions (un-annotated sequence part while other parts of the protein sequence are annotated), dark proteins (proteins without any annotation). Then, to further detect new protein domains, the hydrophobic cluster analysis (HCA) methodology was used. The method was able to extract, in a large number of protein sequences, regions (called here HCA domains) that have a content in hydrophobic residues consistent with a possible ability to fold into compact 3D structures. The remaining parts of these sequences without any HCA domain, or other annotation, do correspond to truly disordered sequences, which appear to be less abundant than expected (4.7% of all of the amino acids). The relative limited amount of sequences used in this study, restricted to Uniprot/Swissprot, probably underestimate the proportion of HCA domains from the dark proteomes sharing no obvious similarity with any other sequence. However, the quality of the sequences from the Uniprot/Swissprot dataset strongly limited the potential bias from artifactual proteins.

It has to be noted that small HCA domains (length < 30 amino acids, 21.8% of the total number of HCA domains) were discarded from the study, but these sequences play important roles, notably on protein-protein or protein-DNA interfaces^14^. Short ordered segments in disordered regions, known as molecular recognition elements (MoREs) or molecular recognition features (MoRFs), have been shown to be key components in protein-protein interactions^48^ and are therefore interesting targets for our understanding of signalling and regulatory pathways. An exhaustive repertoire and description of foldable sequences, also including these short sequences, would thus open new perspectives to fully characterize un-annotated portions of the protein universe.

Analyses of the physico-chemical properties of HCA domains revealed a wide range of sequences and variations inside each initial group of sequences. These variations, in term of physico-chemical properties, reflect the organisation of the protein sequence space continuum, which, in terms of protein sequence foldability, can be described by sequences of globular proteins at one side, and sequences of fully disordered proteins at the other side. Interestingly, each group tends differently toward one side of the range but also displayed some intrinsic variance. Thus, it can be observed that the distributions of physico-chemical properties of PDB region sequences are closer to globular proteins than those of dark protein sequences which are at the opposite side. Moreover, some of the HCA domains, which have a high content in strong hydrophobic amino acids by definition, have however, for the dark proteome, a higher proportion of predicted disorder than annotated domains. This group of sequences can be considered as intermediary in the rating scale of sequence foldability within the protein sequence space and is likely to correspond to pre-molten globule-like extended IDPs^38^.

These results highlight that HCA domains can correspond to structured regions, but also to sequences distant from standard globular protein domains. This last property is likely to correspond to a potential folding capacity of these sequences upon partner binding^14^,^33^, as the HCA domains from the dark sequences have a higher proportion of amino acids to undergo such transition, as predicted using the ANCHOR program. The HCA domains from the dark sequences also have a particular topological organisation of their hydrophobic amino acids, with elongated distances between hydrophobic clusters (corresponding to regular secondary structures), and a lower predicted energy than annotated domains. These features are associated with a lower total content and a large variance in strong hydrophobic residues. Moreover, the relative higher abundance of phosphorylatable residues in the dark HCA domains appears to correlate with the particular behaviour of disordered sequences that undergo disorder-to-order transitions, as phosphorylation is a general mechanism for regulating their functions by providing favourable enthalpy change to the free energy of the binding interaction^49^,^50^,^51^,^52^,^53^,^54^,^55^,^56^, and therefore support their classification as pre-molten globule-like extended IDPs.

Altogether, these results indicate either that un-annotated HCA domains correspond to classes of protein domains distinct from annotated domains or that the methodologies that are classically used to describe domain families are too strict or not enough sensitive to identify some remote members of existing families. A combination of both reasons is also very likely. If novel classes of domains can be defined in this way, this suggests that such sequences may correspond to unseen novel folds, which have been anticipated in some studies^57^. The age of the HCA domains might also partially explain these differences. Indeed, it has been previously shown that the sequences of new-born domain families are also shorter and have a less hydrophobic core but a greater surface area to volume ratio^58^, indicating a difference in organization compared to ancient folds.

Multiple layers of remote homology searches were also conducted here to link a substantial part of the dark proteome sequences to annotated sequences, leading to define different levels of darkness. Only 16286 HCA domains don’t display any significant similarity with any other protein sequences (from which 1470 have no match at all). The remaining HCA domains correspond to either protein sequences that clustered with a relatively high level of similarity using psi-blast or psi-cd-hit or domains with lower similarity levels that either match or not a defined domain in TREMOLO-HCA results. The use of the TREMOLO-HCA approach, combining remote homology searches and domain annotation strategies, might thus be an effective way to map some of the un-annotated sequences of the protein universe.

In conclusion, the presented work provides evidence that the resilience of the dark proteome sequences to be associated with known folds using standard tools is linked both to the particular hydrophobic organization of these sequences and to the limitations of standard tools to detect remote similarity with known protein domain models. Using various annotation resources represents a good methodological choice to reduce potential miss-annotations and to allow the deepest coverage, but new tools are still needed for improving the automatic domain annotation of the always growing number of sequences, particularly in the context of metagenomics analyses^59^.

## Methods

### Dataset

The set of protein sequences considered in this study was extracted from Uniprot/Swissprot, release of November 2015^27^. The Swissprot dataset has the advantage of being meticulously curated with reliable annotations and the proteins of this set are less likely to correspond to artifactual constructs. Another advantage is to allow comparison of our results with the recent study of Perdigão and colleagues^23^, who used the same set of proteins.

The Uniprot/Swissprot dataset is composed of 549 832 proteins. The dataset is separated by kingdoms, with 332 280 protein sequences from Bacteria, 181 584 sequences from Eukaryota, 19 369 sequences from Archaea, and 16 599 sequences from Viruses.

### Annotation

Protein domain annotation was computed for each protein sequence using different resources. Depending on the annotation sources, the protein domain annotations were considered at different levels of reliability. Structural annotations correspond to our “bright” protein universe, *i.e*. the protein domains for which an experimental 3D structure has been solved. This group is later on referred as the PDB group. Annotation from domain HMMs and the Protein Model Portal are considered as the “gray” protein universe, *i.e*. the protein domains with a significant sequence similarity to a known protein domain family. The remained segments of proteins devoid of annotations are labeled as “dark regions” of the protein universe. A protein without any annotation is labeled as a “dark protein”. A sequence of the dark proteome refers to a sequence from either the “dark regions” or the “dark protein” groups.

A correspondence map exists between the protein structure entries of the Protein Databank Bank^26^ and the protein sequences of Uniprot. This correspondence map was used to annotate the Uniprot/Swissprot sequences with highly reliable structural information.

Three different annotation sources were used to annotate the gray protein universe: the ProteinModelPortal (PMP)^60^, the Pfam-A version 28^8^, and the Conserved Domain Database (CDD)^5^. The annotations were performed using the extracted PMP mapping to Uniprot/Swissprot proteins, the scanning tool from the Pfam utilities, and the CDD web server using default parameters. Annotations of the PDB group were extracted from the PDB/Uniprot mapping available on the Uniprot website.

### Physico-chemical features

Several statistical analyses were conducted after annotation. For the different groups considered here, comparisons were performed to highlight possible differences at the sequence level through evaluation of the amino acid composition. A particular attention was also given to the physico-chemical properties of the studied sequences and to their characterization through hydrophobic cluster analysis (HCA).

#### Protein sequence description

The sequences from dark regions and dark proteins were investigated using the HCA methodology^14^,^25^,^28^,^29^ which is presented in Fig. S13 in more details. Particularly, long segments with high density in hydrophobic clusters (typical of folded domains) were searched using the SEG-HCA software^14^. A minimal size threshold of 30 residues was applied to consider the segments found by SEG-HCA as potential foldable domains that can behave as autonomous folding units. Thus, the dark regions were separated into two different groups, likely foldable regions (presence of a hydrophobic core) and too short or too hydrophilic segments. The likely foldable sequences are referred to as the HCA domains.

These sequences were compared to HCA domains extracted from the sequences of the PDB regions and gray regions in order to understand why these regions still don’t have any domain annotation. This step allows a fair comparison between groups insofar as HCA domains have previously been compared to annotated domains and seen to cover them very well^14^.

Different descriptors were compared between sequences of each group: domain lengths, amino acid frequencies, content in hydrophobic amino acids, intrinsic disorder (estimated through the consensus field of the MobiDB^34^ database), sites to undergo disorder-to-order transition upon binding (predicted by ANCHOR^35^,^36^), and the percentage of residues predicted in transmembrane regions (computed from the transmembrane field of the Uniprot sequence description). As the domain detection by SEG-HCA heavily relies on the presence of strong hydrophobic amino acids (V, I, L, F, M, Y, W), a topological analysis of the hydrophobic clusters was performed. To that aim, the distances between hydrophobic clusters were calculated (between the last amino acid of a first cluster and the first amino acid of a second cluster, but also between the first amino acid of the same first cluster and the last amino acid of the same second cluster) and used as a proxy for hydrophobic cluster topology.

#### Foldability of sequences from the dark proteome

Domains detected by SEG-HCA have the general characteristics of folded domains. To go further in the comparison of the sequences of the different groups, simulation using HP-lattice models were conducted using the HPstruct tool from the CPSP package^61^. Difference in energy relative to the composition in hydrophobic amino acids could indicate a general difference in topology that may explain the lack of annotation for the SEG-HCA domains included in the dark proteome. 5000 protein sequences of each group were randomly chosen and drawn, as a comprehensive calculation is not possible, due to computing limitations.

#### Statistical tests

Distributions were compared using the two sample Kolmogorov-Smirnov test as the distributions do not appear following a Gaussian law. However, hypothesis testing is very sensitive to even small deviations when comparing large datasets as here. Therefore, our analyses focused on the observed differences between the groups and their interpretations, rather than on the computed p-values. However, some p-values are given when judged necessary.

### Similarity searches

Absence of annotations for the domains of the dark proteomes described as foldable by the SEG-HCA software may originate from multiple causes. A deeper investigation of the potential similarities that the dark sequences may share with other sequences was therefore conducted.

Several methodologies described in, Fig. S14, were deployed to further characterize HCA domains belonging to the dark regions and to the dark proteins.

First, as a general method to eliminate potential miss-annotations of sequences within the dark proteome, PSI-BLAST^62^ searches were performed using the HCA domain sequences of the dark proteome as queries against sequences of the gray and PDB groups. HCA domain sequences with matches characterized by an E-value below 0.001 and a coverage greater than 80% were considered as miss-detected known domains. Second, the remaining HCA domains from the dark region and dark protein sequences were then pulled together and a psi-cd-hit^63^,^64^ analysis was performed (local alignment parameters with successive cutoffs of 90, 60 and 30 sequence identity). This method was used to evaluate the number of potential, still undescribed families within the dark regions.

Finally, HCA domain sequences which do not belong to any cluster were then further analyzed using TREMOLO-HCA, a tool designed to unravel remote relationships that HCA domains sequences of the dark proteome may share with other proteins^30^. Briefly, TREMOLO-HCA performs a HHblits search^39^ followed by a CDD annotation, thus associating remote homology detection and domain context. In this study, the uniprot_20 database (release of 2015) was used as the target database. Regarding TREMOLO-HCA results, the HCA domains were separated into different categories: HCA domains having a significant match to a target HHblits (e-value < 0.001) and for which the boundary of the match corresponds to a domain (coverage > 80%), HCA domains having a significant match but for which the boundary does not entirely cover a domain (0% < coverage <=80%t), HCA domains with a significant match but to which no known domain is associated, and HCA domains without significant match (HHblits e-value > 0.001) or without any match. HCA domains having a significant match which does not correspond to a known domain were clustered together using MCL^65^. First, a graph was constructed based on common target matching, *i.e*. if two HCA domains matches the same target sequence, with the coverage between the target matches being greater or equal to 50% of the longest match, an edge was created between the two HCA domains. The graph was then clustered using MCL inflation parameter of 2.5.

## Additional Information

**How to cite this article**: Bitard-Feildel, T. and Callebaut, I. Exploring the dark foldable proteome by considering hydrophobic amino acids topology. *Sci. Rep.* **7**, 41425; doi: 10.1038/srep41425 (2017).

**Publisher's note:** Springer Nature remains neutral with regard to jurisdictional claims in published maps and institutional affiliations.

## Supplementary Material

Supplementary Information

## Figures and Tables

**Table 1:** Psi-cd-hit results.

Cluster size	Number of sequences	Number of clusters by sequence length					
		10–59	60–149	150–499	500–1999	2000+	total
1	67217	37381	22662	6663	509	2	67217
2–4	51972	8548	8585	3365	380	6	20884
5–9	22104	830	1424	1078	200	5	3537
10–19	9191	130	236	290	73	2	731
20–49	4355	32	34	51	37	1	155
50–99+	1389	1	6	2	10	2	21
Total	156228	46922	32947	11449	1209	18	92545

The table shows the number of sequences belonging to a specific cluster size and the number of clusters for a specific sequence length (based on the representative sequence of each psi-cd-hit cluster).
