# Supplementary material for: Exploring the dark foldable proteome by considering hydrophobic amino acids topology
Source: Sci Rep. 2017 Jan 30;7:41425. doi: 10.1038/srep41425 (PMC5278394; doi:10.1038/srep41425)
Supplement: Supplementary Information [file srep41425-s1.pdf]

Supplementary Material

Exploring the foldable dark proteome by considering hydrophobic amino acids topology

Tristan Bitard-Feildel, Isabelle Callebaut

Supporting Information

Fig. S1 illustrates the percentages of sequences with or without domain annotations in the four different kingdoms of life. Depending on the annotation or un-annotation type, the sequences are labeled according to four different categories: a protein without any annotation is labeled as a dark protein, an un-annotated region of a protein as a dark region, a region corresponding to a Pfam or a CDD domain as a gray region, and a region corresponding to a PMP or a mapped PDB sequence as a PDB region.

Fig. S2 shows the percentages of amino acids covered by HCA domains for each of the categories considered here (dark proteins, dark regions, gray regions, PDB regions). The raw numbers are presented in **Table S1**. Sequences from gray and PDB regions have a high number of amino acids belonging to HCA domains (91.41% in Eukaryota, 94.97% in Bacteria, 96.05% in Archaea and 91.79% in Viruses for sequences of gray regions, and 88.59% in Eukaryota, 93.98% in Bacteria, 96.33% in Archaea and 89.71% in Viruses for sequences of PDB regions), in agreement with their general globular character. The percentage of residues in HCA domains varies between kingdoms regarding the dark regions and dark proteins groups. Archaea have far more covered dark protein sequences (94.73%) than bacteria (88.97%) and viruses (89.29%), which have themselves a higher coverage than Eukaryotes (77.99%). Regarding dark regions, the levels of amino acid coverage by HCA domains are comparable, except for viral sequences, which are more covered (75.36%, 71.87%, 79.36% and 88.29% respectively for Eukaryota, Bacteria, Archaea and Viruses groups). The general proportion of dark sequences by kingdom should also been taken into account when considering these percentages of coverage. Hence, eukaryotic sequences, for which the amount of dark sequences is higher, have a higher proportion of amino acids not covered by HCA domains. This behavior should be expected as non-HCA domains correspond mainly to disordered sequences [1].

Fig. S3 shows the distribution of the domain lengths for the four categories of sequences. Domains from the dark regions and dark proteins are shorter than domain from gray and PDB regions. The mode of the distributions are however relatively similar.

Fig. S4 shows the fold change ratio of the percentage mean values in the 20 amino acids between the sequences of the PDB regions and of the other groups of sequences. No large variation is observed, the maximal fold change being observed for serine between the sequences of the dark regions and the PDB regions. However, the fold change value is rather small, 0.36, and does not reflect any significant change in the amino acid composition of the sequences.

Fig. S5 shows the distributions of the amino acid frequencies within each group of sequences. The means of distributions are very similar but some strong variation can be noted in the variance for some amino acids.

Fig. S6 shows the distribution of the hydrophobic cluster lengths within the four different groups of sequences. The shapes of the distributions are very similar but there are higher numbers of smaller clusters in the sequences of the dark regions and larger clusters in the sequences of the dark proteins.

Fig. S7 shows the prediction sites that undergo disorder-to-order transition upon binding (disordered binding regions), as predicted by the ANCHOR program, of the different groups of sequences. **Fig. S7a** displays the distribution of the proportion of these sites for the four initial groups of sequences (PDB regions, gray regions, dark regions, dark proteins). A higher proportion of predicted “disordered binding regions” is observed for the

protein sequences of the dark proteomes, particularly for the sequences of the dark regions. **Fig. S7b** displays the distributions of the predicted "folding upon binding" activity for the HCA domains sequences after clustering according to TREMOLO-HCA results. HCA domains with the lowest levels of detected remote homology are characterized by higher content of predicted "disordered binding regions".

Fig. S8 shows the d1 and d2 distances, estimating the degree of separation between hydrophobic clusters, of random sequences with various sizes (see **Fig. S13** for the definition of these distances). The d1 and d2 distances increase with the length of the randomly generated sequences. In contrast, **Fig. 3** of the main document shows the d1 and d2 distances for the four different groups of actual sequences. In that case, the shorter sequences from the dark regions and the dark proteins have larger d1 and d2 distances than the longer sequences of the gray and PDB regions.

Fig. S9 to **S12** show four examples of HCA domains used as TREMOLO-HCA queries and each corresponding to one of the four types of classification applied considering the TREMOLO-HCA results. **Fig. S9** corresponds to a HCA domain for which a sequence match was found with a domain from a database. **Fig. S10** shows a HCA domain for which only a partial match to a domain from a database was found. **Fig. S11** displays a HCA domain present in multiple sequences, but not corresponding to any domain annotation. **Fig. S12** shows two HCA domains for which no sequence with significant similarity was found in the uniprot_20 database.

HCA methodology

Fig. S13 explains the principles of the Hydrophobic Cluster Analysis (HCA) approach and how are generated the HCA plots shown in Supplementary **Fig. S9 to S12**. From an original 1D amino acid sequence (panel A), a 2D plot is created (panel D) by writing the amino acid sequence along an α-helix (panel B) and cutting it along its horizontal axis. The helix forms in a two dimensional space a plane (panel C) on which every line of amino acids corresponds to a helix turn. The plane is duplicated and the hydrophobic clusters are defined by joining contiguous strong hydrophobic amino acids (V,I,L,F,M,Y,W). This set of hydrophobic amino acids was determined after analyses of the relationships between hydrophobicity, regular secondary structure elements, and solvent accessibility [2]. All of the seven amino acids considered in the HCA alphabet display a strong hydrophobic character, particularly when directly estimated within the context of globular proteins [3,4].

Workflow

Fig. S14 illustrates the full, general workflow used in this study (for details of each step, please refer to the Methods section). Briefly, the methodology used the protein sequences from Uniprot/Swissprot (release of November 2015 (i)). For each sequence, domain annotation was performed using various sources: PDB, PMP, CDD, Pfam (ii). PDB and PMP annotations were extracted from their respective mapping with Uniprot sequences, Pfam annotation was performed using the Pfam scan tool (using default parameters and version 28 of the Pfam-A database), and the CDD annotation was computed using the CDD web server with default parameters. Parts of protein sequences corresponding to PDB or PMP annotations were classified as belonging to the known protein universe for which a 3D structure has been solved ("PDB regions" classification) (iii). Parts of sequences matching a domain database model (CDD/Pfam) were classified as the "gray regions" of the protein universe (iii). Un-annotated parts of protein sequences were either classified as "dark regions" or "dark proteins" if the corresponding proteins had another part of their sequences already annotated in the PDB region or gray region groups, or if the proteins were completely un-annotated, respectively (iv).

The Seg-HCA tool was then used to detect potential foldable regions inside the sequences (v) of the dark regions and dark proteins groups (vii), as well as those of the gray and PDB regions (vi). These HCA domains were later considered to derive physico-chemical properties of the sequences of the two groups, which were then compared.

Furthermore, several tools were applied to characterize sequences of the dark regions and dark proteins and to separate sequences of the dark proteome sharing various degrees of similarities with other sequences from sequences truly remaining without known information. First, PSI-BLAST searches (viii) were performed using as queries HCA domain sequences of the dark regions and dark proteins (vii) against the sequences of the PDB and gray regions (vi). Sequences without any significant matches (ix) were selected and clustered using psi-cdhit (local

alignment parameters with successive cut-offs of 90, 60 and 30 % of sequence identity) (x). Non-clustered sequences of the dark proteome (xi) were then selected and a Tremolo-HCA analysis was performed for each of them (xii). The protein sequences used as queries were grouped into four categories regarding the Tremolo-HCA results (xiii): (a) the sequences matching and covering (overlap > 80%) a domain within one of the Tremolo-HCA/HHblits hits, (b) the sequences matching but only partially covering (coverage < 80%) a domain within one of the Tremolo-HCA/HHblits hits, (c) the sequences matching a Tremolo-HCA/HHblits hit but without any domain associated with this hit, (d) the sequences which do not significantly align with any other sequence.

Supporting Information – Figures

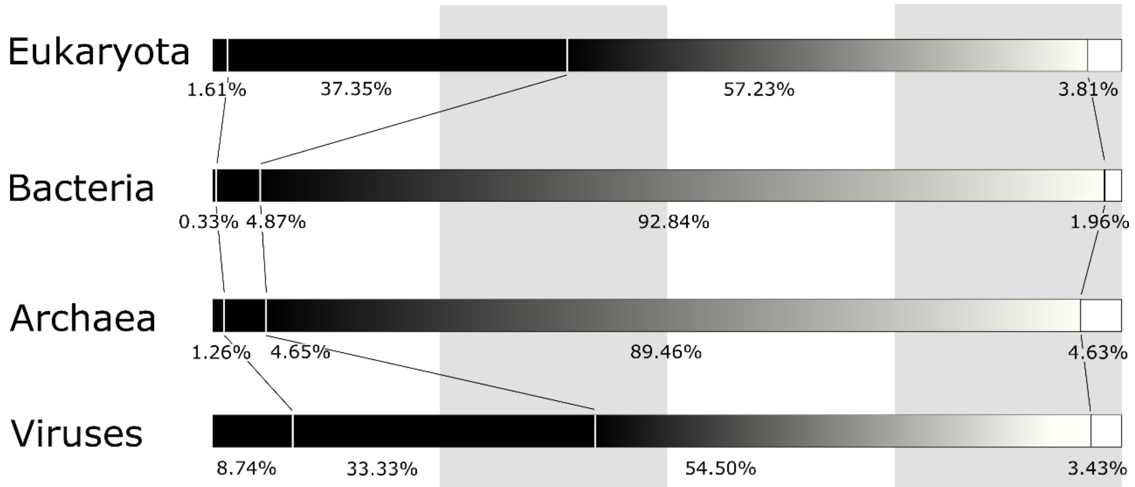

Figure S1- Percentages of annotated/un-annotated sequences in the proteomes of the four kingdoms of life. *From left to right: dark proteins (proteins without any annotation), dark regions (un-annotated regions of proteins), gray regions (regions of proteins annotated by Pfam or CDD), PDB regions (PMP and Uniprot/PDB mapped regions).*

Kingdom	Percentage relatively	Dark proteins		Dark regions		Gray regions		PDB regions	
		No HCA domains	HCA domains	No HCA domains	HCA domains	No HCA domains	HCA domains	No HCA domains	HCA domains
**Eukaryota**	to kingdom	0.35	1.26	9.20	28.15	4.92	52.31	0.44	3.38
	to group	22.01	77.99	24.64	75.36	8.59	91.41	11.41	88.59
**Bacteria**	to kingdom	0.04	0.29	1.37	3.50	4.67	88.17	0.12	1.84
	to group	11.03	88.97	28.12	71.87	5.03	94.97	6.02	93.98
**Archaea**	to kingdom	0.07	1.19	0.96	3.69	3.54	85.93	0.17	4.46
	to group	5.27	94.73	20.74	79.36	3.95	96.05	3.66	96.33
**Viruses**	to kingdom	0.94	7.81	3.90	29.43	4.47	50.03	0.35	3.08
	to group	10.71	89.29	11.71	88.29	8.21	91.79	10.29	89.71
**Data from Perdigão et al (2015)**									
**Eukaryota**		15%		29%		52%		4%	
**Bacteria**		5%		8%		85%		2%	
**Archaea**		6%		8%		82%		4%	
**Viruses**		28%		26%		42%		4%	

Table S1- Amino acid coverage by foldable (HCA) domains for each sequence group in the different kingdoms. *For comparison purposes, the percentages calculated by Perdigão et al (2015) [5] (considering only structural information – see main text) are recalled at bottom.*

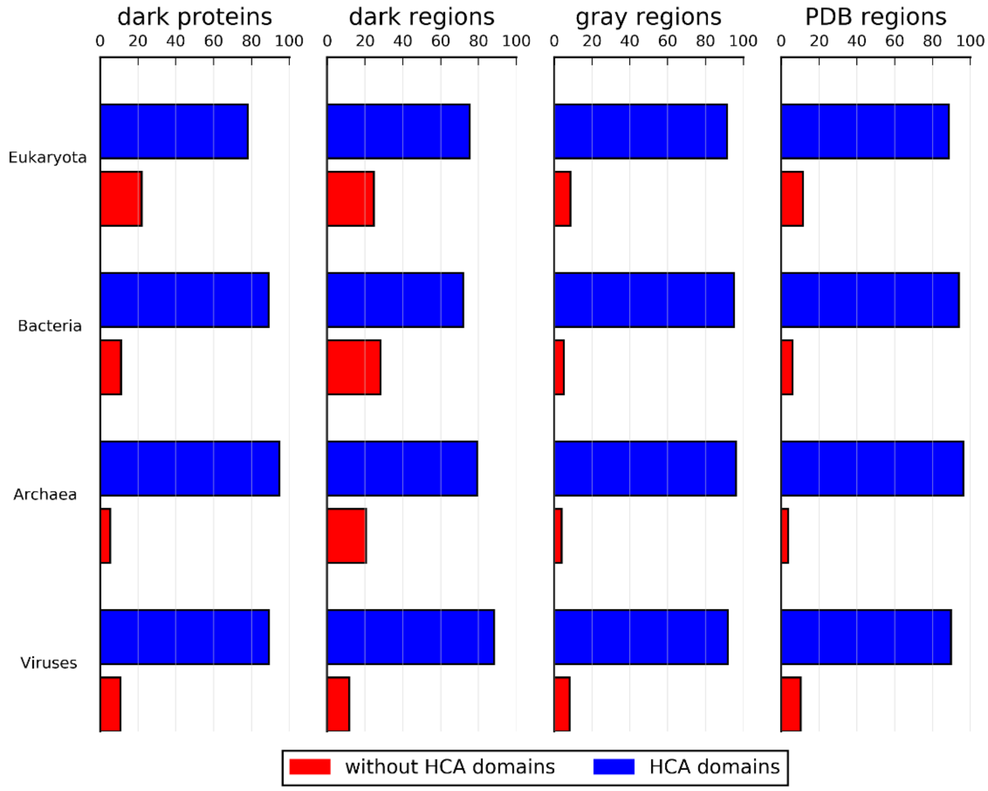

Figure S2 - Coverage in foldable (HCA) and non-foldable (without HCA) domains in the four Kingdoms of life*. Foldable domains are delineated using SegHCA, within the sequences of each protein group. Sequences from gray and PDB regions are particularly well covered as they correspond to known protein domains or folds. The un-covered parts correspond to regions without enough organized hydrophobicity and therefore probably disordered.*

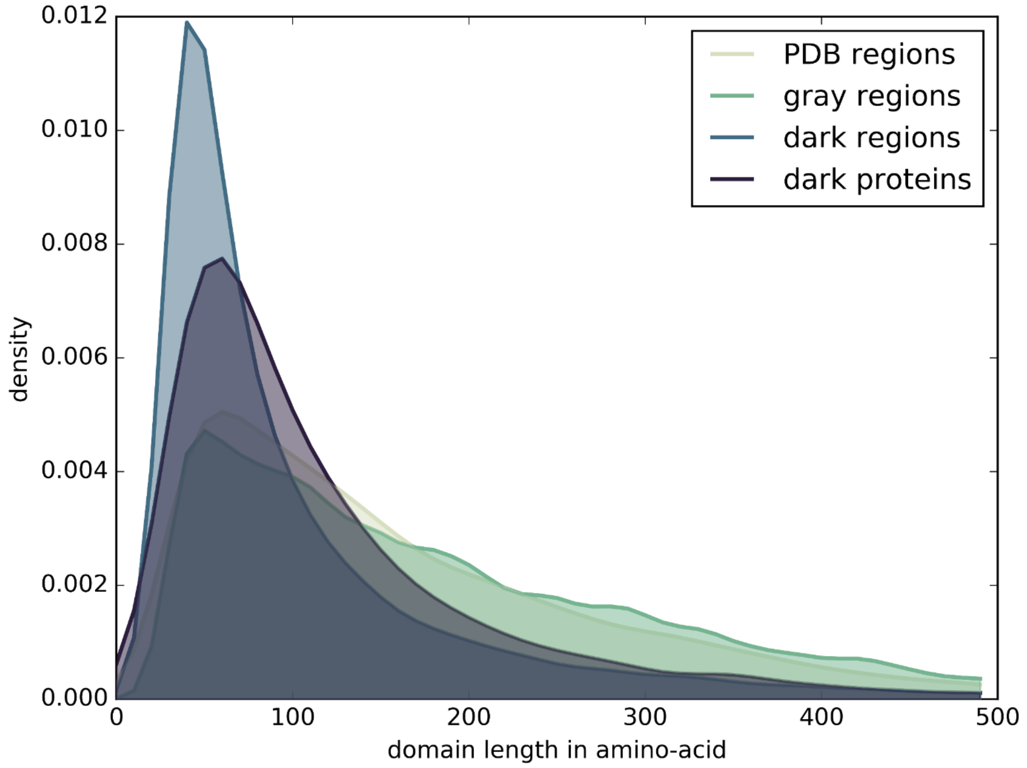

Figure S3 - Domain length distributions for the different groups of sequences. *Mean lengths (in amino acids) for PDB regions: 173.65, for gray regions: 195.47, for dark regions: 114.29, for dark proteins: 129.71. The shortest sequences belong to the dark region group. This group is composed of un-annotated parts of protein sequences that have otherwise another part annotated.*

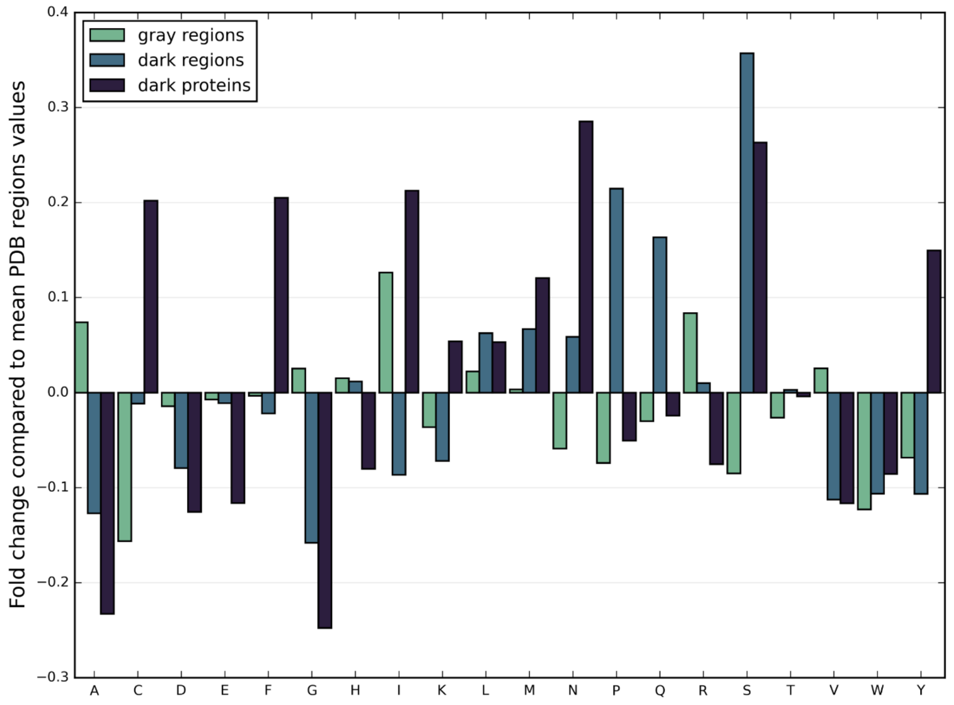

Figure S4 - Fold change ratio between sequences of the PDB regions and of the three other groups. *The ratio is computed using the mean percentages in the 20 amino acids found in sequences of each group. A larger difference in positive fold change between the dark proteins and PDB regions groups can be observed for the amino acids C, F, I, M, N, S, Y as well as negative fold change for the amino acids A, G, V. Comparing dark regions with PDB regions, the amino acids P, Q, S are more present and the amino acids A, G and V are less observed in dark regions. The gray regions group shows less marked differences but I is more present and C and W are less frequent in gray regions than in PDB regions.*

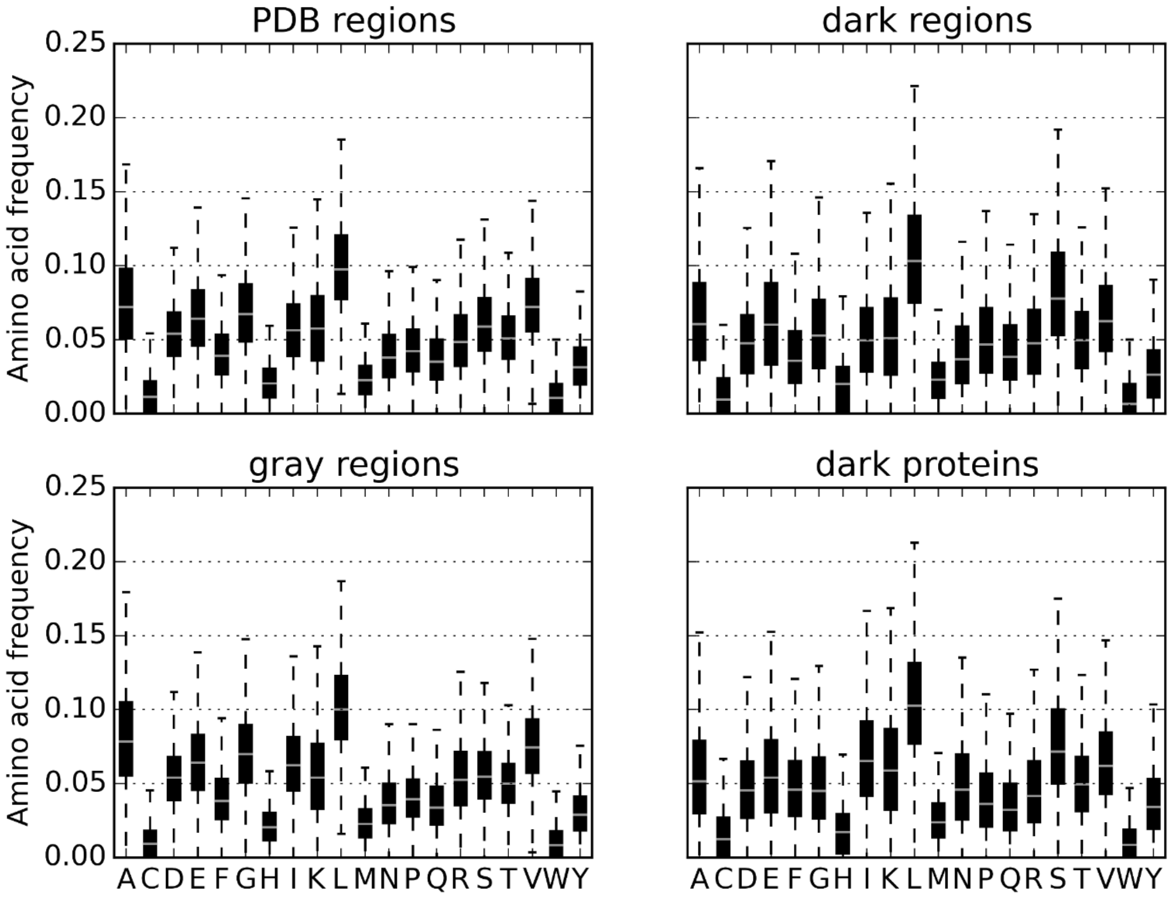

Figure S5 - Boxplot of the mean amino acid composition per sequence. *The distributions are computed for the four different groups of protein sequences. The distributions of amino acid percentages per sequence show a large dispersion, indicating strong variation between protein sequences of the same groups.*

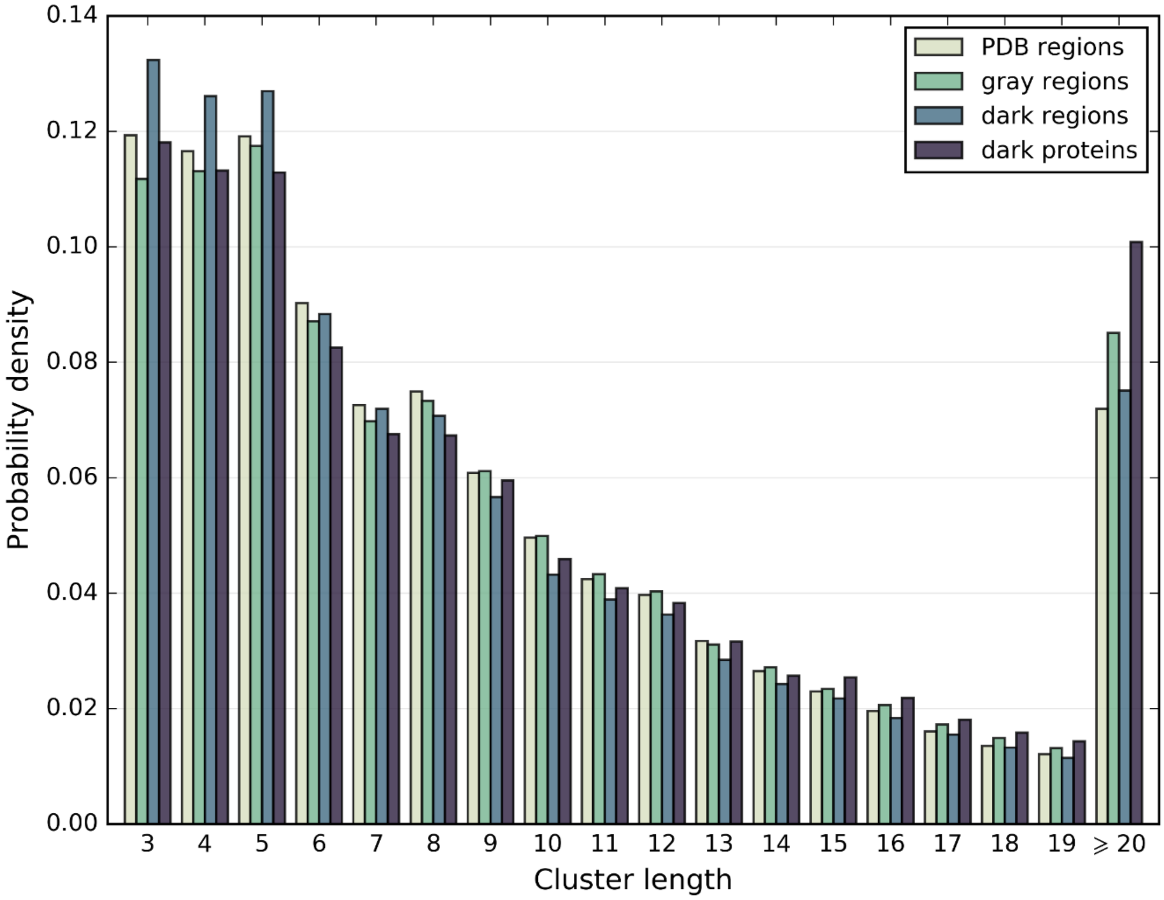

Figure S6 - Distributions of the hydrophobic cluster lengths (in amino acids) in foldable (HCA) domains. *Dark region sequences have more small hydrophobic clusters than the other groups and dark proteins sequences have more long hydrophobic clusters, particularly hydrophobic clusters longer than 20 amino acids.*

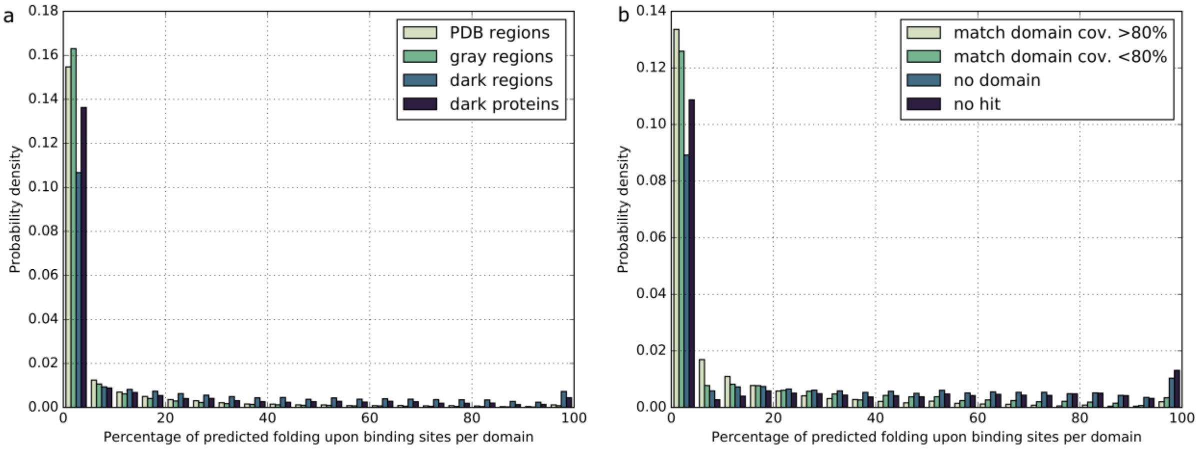

Figure S7 – Percentage of sites with disorder-to-order transition upon binding predicted by ANCHOR. *Panel a shows the distribution of the percentage of sites predicted to fold upon binding for the HCA domain sequences of the group initial groups. Distributions of Panel b are computed for the HCA domains clustered according to the TREMOLO results of the dark sequences.*

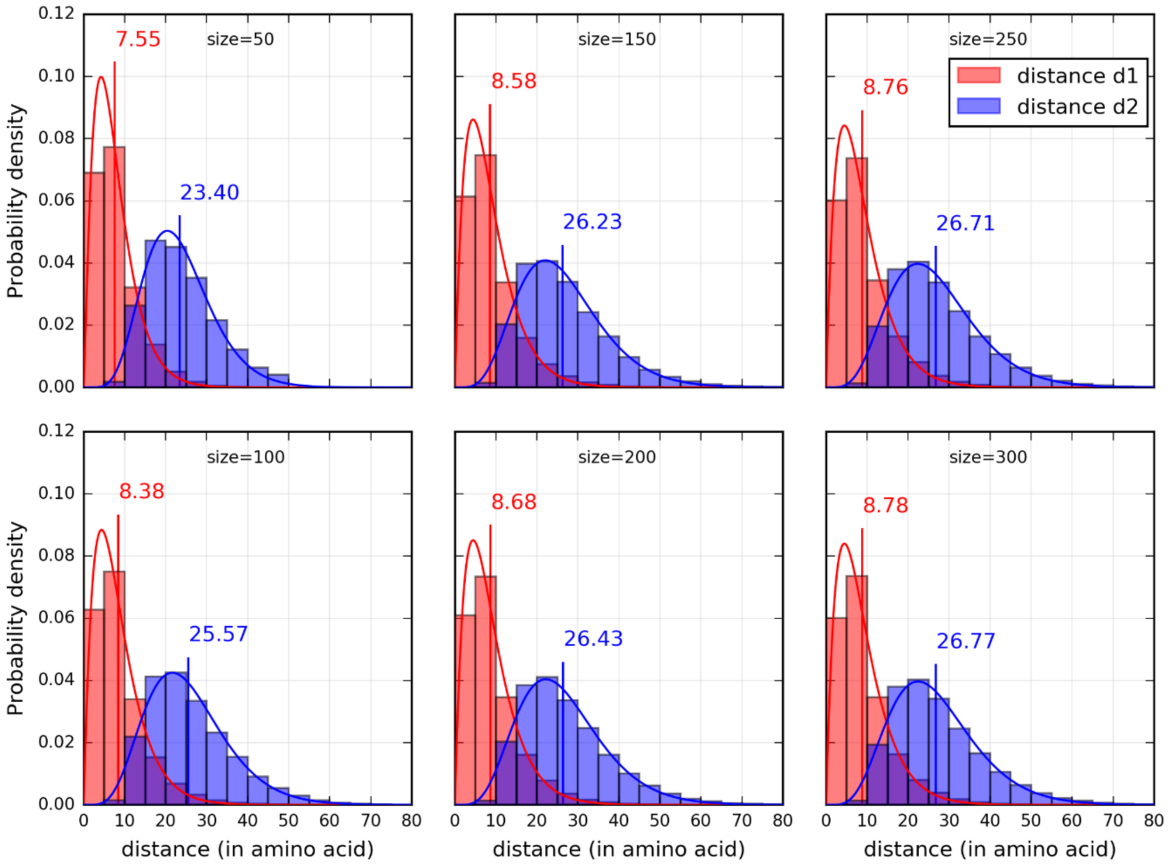

Figure S8 - Distances between hydrophobic clusters for various sizes of random sequences. *The distances are computed between the last amino acids of a hydrophobic cluster and the first amino acids of the following hydrophobic cluster (distance d1) and between the first amino acids of a hydrophobic cluster and the last amino acids of the following hydrophobic cluster (distance d2) for randomly generated sequences, with amino acid mean frequencies taken from the PDB sequences. See Fig. S13 for an illustration of the d1 and d2 distances. The d1 and d2 distance distributions are size dependent and longer sequences have distributions shifted towards longer distances. These distributions should be compared to the d1 and d2 distributions of the four different groups of actual sequences analysed in the main manuscript and for which an opposite trend is observed: sequences from dark regions and dark proteins have longer distances but are also shorter (Fig S5) than sequences from PDB and gray regions.*

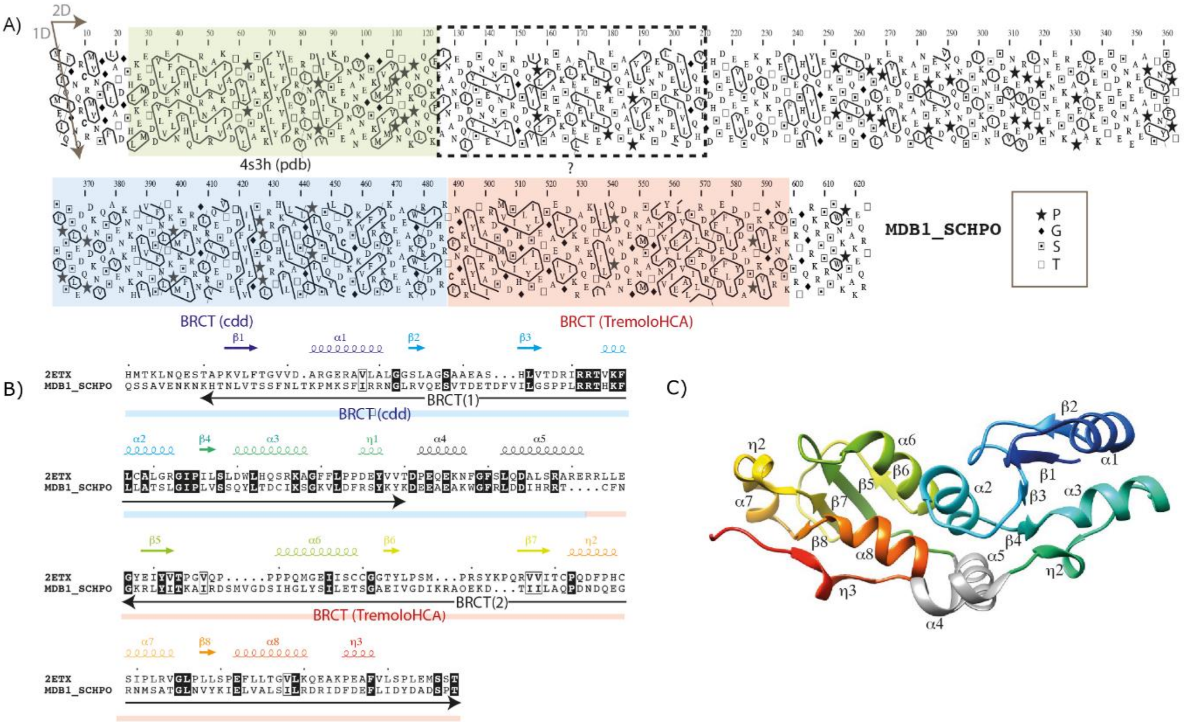

Figure S9 - Detection of a hidden BRCT domain within the C-terminal HCA domain of fission yeast MDB1 protein. *Panel A displays the domain architecture reported on the HCA diagram of the query Uniprot protein sequence MDB1 (O14079). One domain was annotated through PDB mapping, one through CDD search and two domains were found by Seg-HCA in the dark proteome (an unknown domain (dashed box) downstream of the FHA domain (pdb 4s3h), and the hidden BRCT domain downstream of the BRCT domain found after CDD annotation). Panel B shows the alignment between the HCA domain sequence query (MDB1_SCHPO) and the target sequence hit found in the TREMOLO-HCA results (human ortholog MDC1, PDB 2etx). Panel C presents the ribbon representation of the 3D structure human ortholog MDC1 BRCT tandem repeat (PDB code 2etx) and the positions of the different secondary structure elements reported on panel B.*

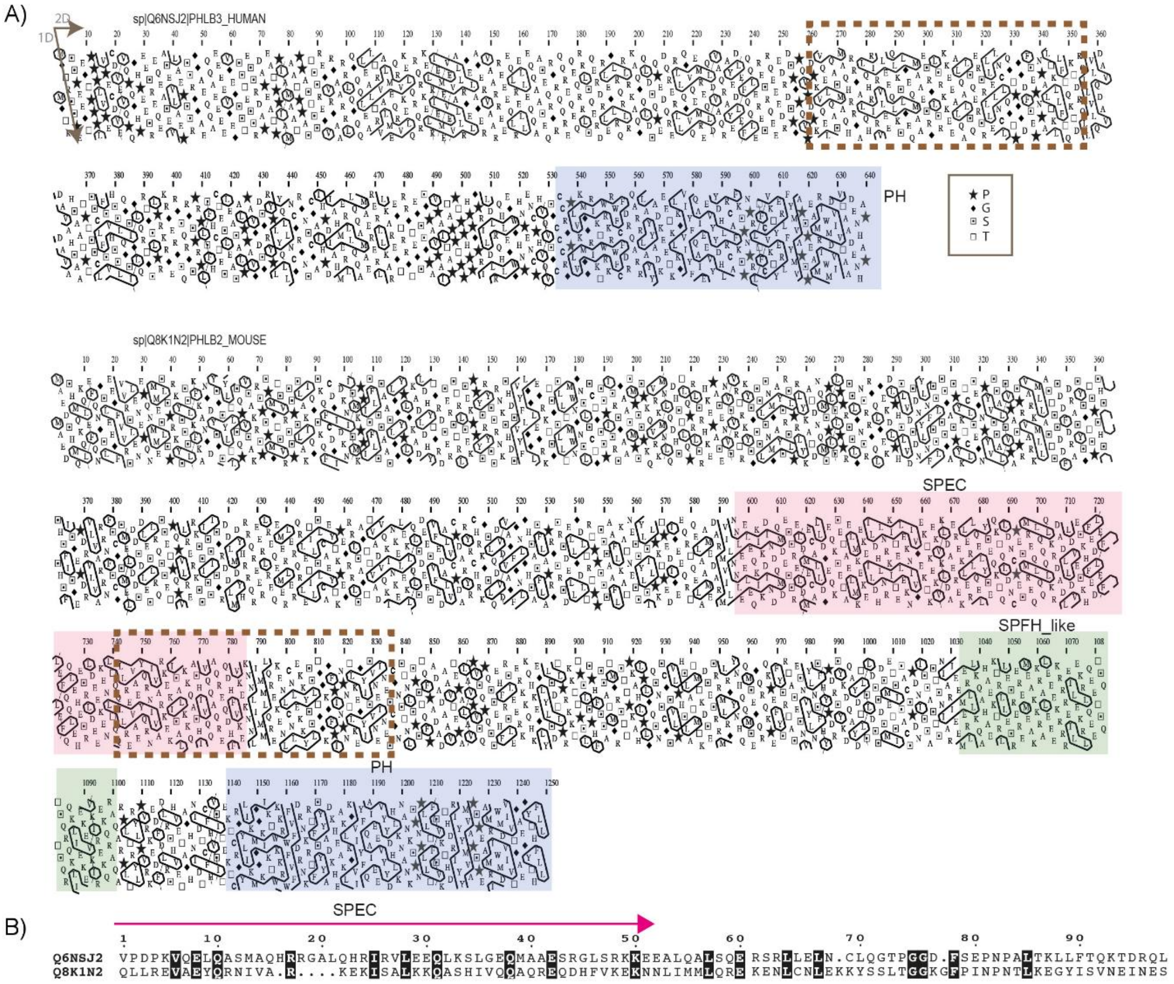

Figure S10 - Partial match of a HCA domain sequence from the PHLB3 protein (Uniprot identifier Q6NSJ2) with a TREMOLO-HCA target. *The TREMOLO-HCA hit partially corresponds to an annotated domain (annotation performed using CDD). Panel A shows the HCA plots of the two proteins (query on top, with the HCA domain in a dashed box, target on bottom) with the annotation performed using CDD. Panel B shows the amino acid alignment between the HCA domain query (top) and the TREMOLO-HCA target (bottom). The position of the partially matched domain (SPEC domain) is delineated with an arrow (CDD accession numbers: PH-like: cl02488, SPEC: cl19107, PH: cl17171).*

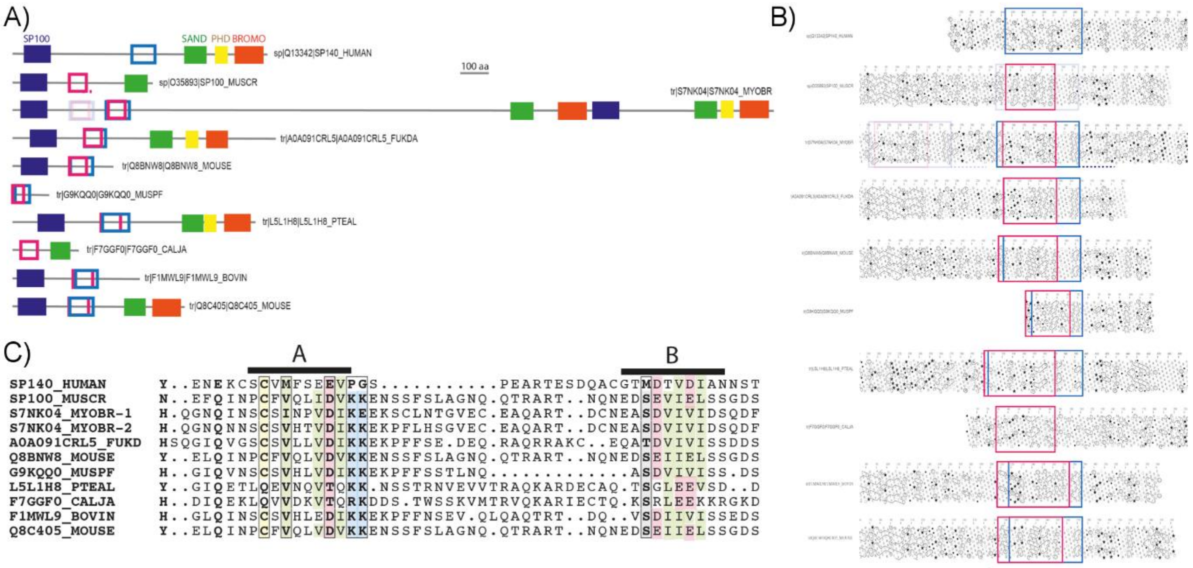

Figure S11 - HCA domains matching similar targets without domain annotation. *Two HCA domains, from protein sequences with Uniprot identifiers Q13342 (SP140_HUMAN) and O35893 (SP100_MUSCR), were clustered together based on the similarity of their sets of hits computed by TREMOLO-HCA. Panel A shows the two query proteins (top first two proteins) and their common targets with corresponding domain annotations. The HCA domains are delineated by blue (for protein Q13342) and pink (for protein O35893) boxes. Panel B shows the HCA plots of the proteins and the positions of the un-annotated domains. Panel C displays a part of the multiple sequence alignment (with two conserved motifs A and B) made using MAFFT* [6] *and manually improved after consideration of the hydrophobic cluster conservation.*

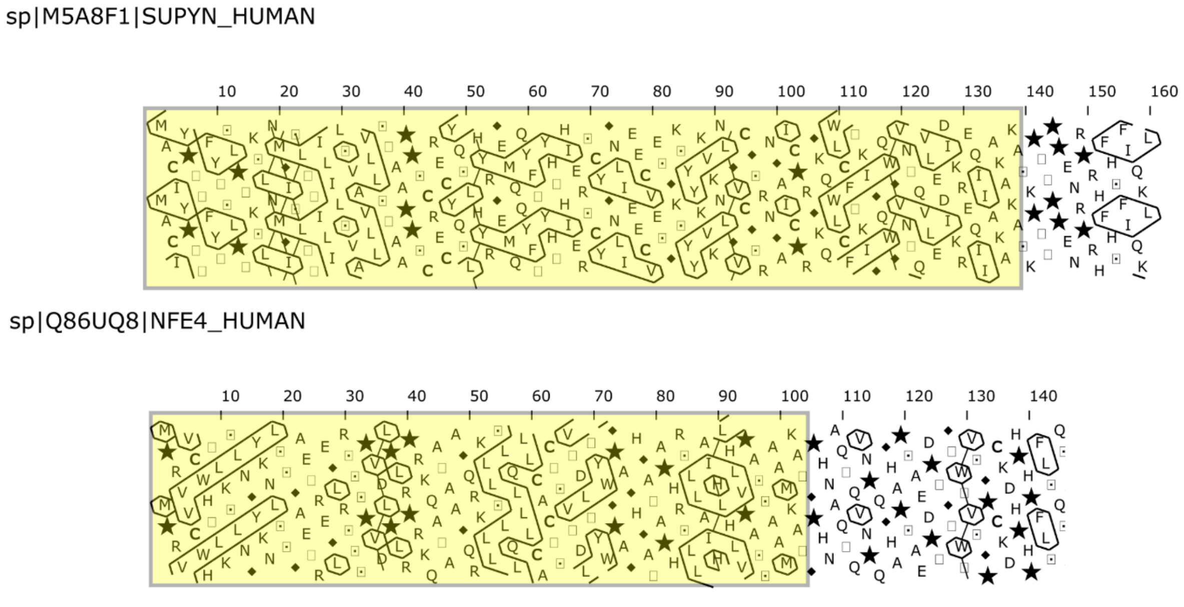

Figure S12 - Two HCA domains for which no significant remote homologs were found. *The two domains are not related to each other and are presented to illustrate true dark protein sequences. Note, in the first sequence, the presence of a large hydrophobic cluster (between amino acids 20 and 36), typical of a transmembrane segment, and in both sequences, the abundance of cysteine residues.*

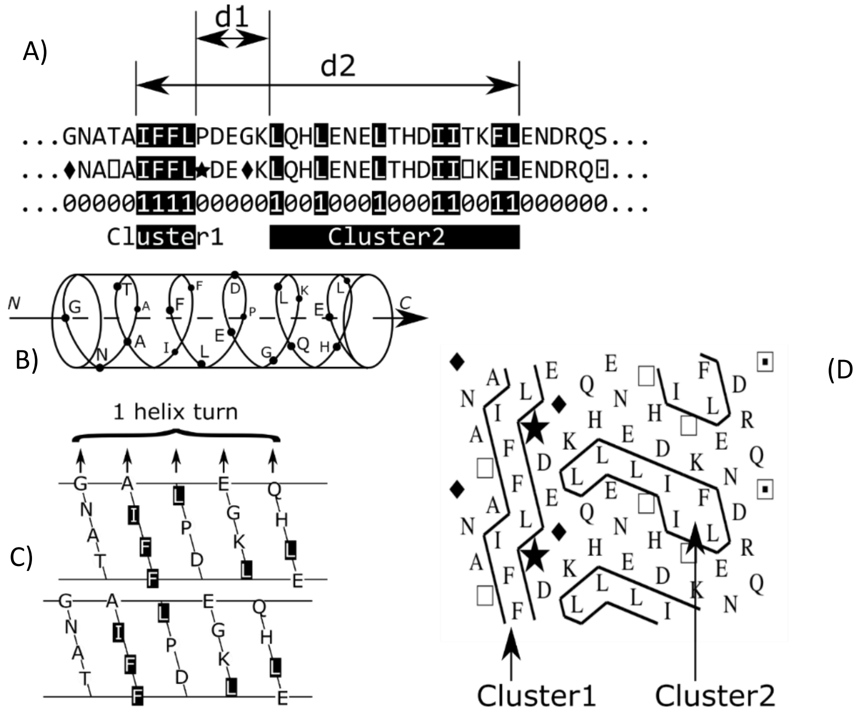

Figure S13- **Principle of the HCA plot.** Panel A, the protein sequence (1D), in which hydrophobic amino acids are represented as white letters, is written on an α-helix, displayed on a cylinder (panel B). This one is cut along the horizontal axis and unrolled, in order to get the full environment of each amino acid, as it exists on the 1D sequence (panel C). Strong hydrophobic amino acids (V, I, L, F, M, Y, W) are encircled and their contours are joined (panel D), forming clusters. Horizontal and vertical clusters are mainly associated with alpha helices and beta strands, respectively [7,8]. The d1 and d2 distances shown in panel A allows the estimation of the separation of hydrophobic clusters.

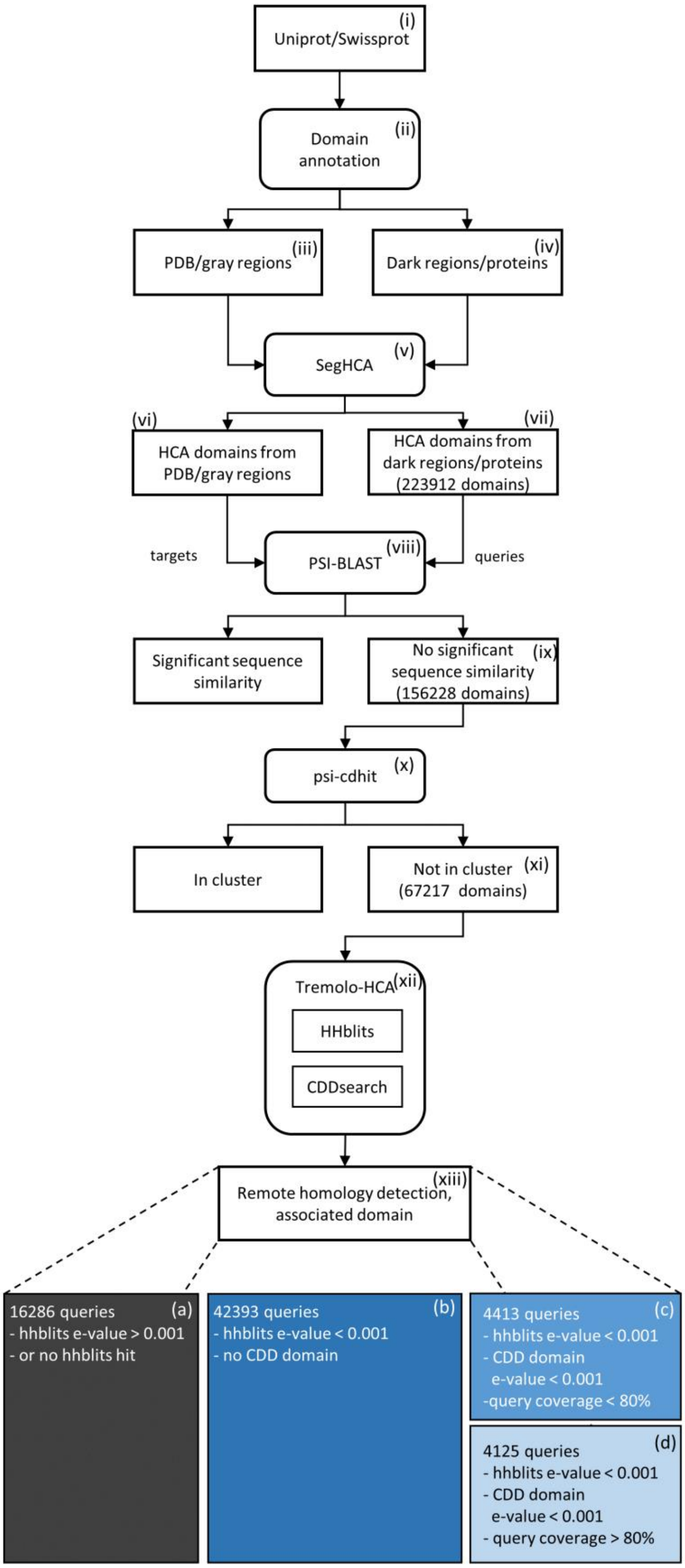

Figure S14 - Full general workflow used to classify the sequences from the dark universe.

References

1. Faure, G. & Callebaut, I. Comprehensive Repertoire of Foldable Regions within Whole Genomes. *PLoS Comput. Biol.* **9,** e1003280 (2013).
2. Woodcock, S., Mornon, J. P. & Henrissat, B. Detection of secondary structure elements in proteins by hydrophobic cluster analysis. *Protein Eng.* **5,** 629–35 (1992).
3. White, S. H. & Wimley, W. C. Membrane protein folding and stability: Physical principles. *Annu. Rev. Biophys. Biomolec. Struct.* **28,** 319–365 (1999).
4. Moret, M. A. & Zebende, G. F. Amino acid hydrophobicity and accessible surface area. *Phys. Rev. E - Stat. Nonlinear, Soft Matter Phys.* **75,** (2007).
5. Perdigão, N. *et al.* Unexpected features of the dark proteome. *Proc. Natl. Acad. Sci.* **112,** 15898–15903 (2015).
6. Katoh, K. & Standley, D. M. MAFFT multiple sequence alignment software version 7: Improvements in performance and usability. *Mol. Biol. Evol.* **30,** 772–780 (2013).
7. Eudes, R., Le Tuan, K., Delettré, J., Mornon, J.-P. & Callebaut, I. A generalized analysis of hydrophobic and loop clusters within globular protein sequences. *BMC Struct. Biol.* **7,** 2 (2007).
8. Rebehmed, J., Quintus, F., Mornon, J.-P. & Callebaut, I. The respective roles of polar/nonpolar binary patterns and amino acid composition in protein regular secondary structures explored exhaustively using hydrophobic cluster analysis. *Proteins Struct. Funct. Bioinforma.* **84,** 624–638 (2016).
